# Artificial intelligence (AI) psychosis: mechanisms, clinical risks and safety considerations in generative AI chatbots

**DOI:** 10.1192/bjo.2026.12021

**Published:** 2026-06-11

**Authors:** Lotenna Olisaeloka, John-Jose Nunez, Daniel V. Vigo, Raymond Ng

**Affiliations:** Psychiatry, University of British Columbia, Vancouver, British Columbia, Canada; Computer Science, University of British Columbia, Vancouver, British Columbia, Canada

**Keywords:** Artificial intelligence psychosis, generative artificial intelligence, artificial intelligence chatbots, mental health artificial intelligence, artificial intelligence safety

## Abstract

As generative artificial intelligence chatbots become embedded in everyday life, concerns about their psychological risks are growing. Emerging reports describe cases of artificial intelligence-induced or -associated psychosis (hereafter artificial intelligence (AI) psychosis) in which intensive chatbot use is associated with delusional thinking patterns. This paper proposes a provisional mechanism wherein baseline user vulnerabilities and engagement patterns interact with generative artificial intelligence characteristics, such as sycophancy and hallucination, contributing to delusional ideation. It subsequently outlines clinical, design and regulatory strategies that may help mitigate risks.

**Figure:**
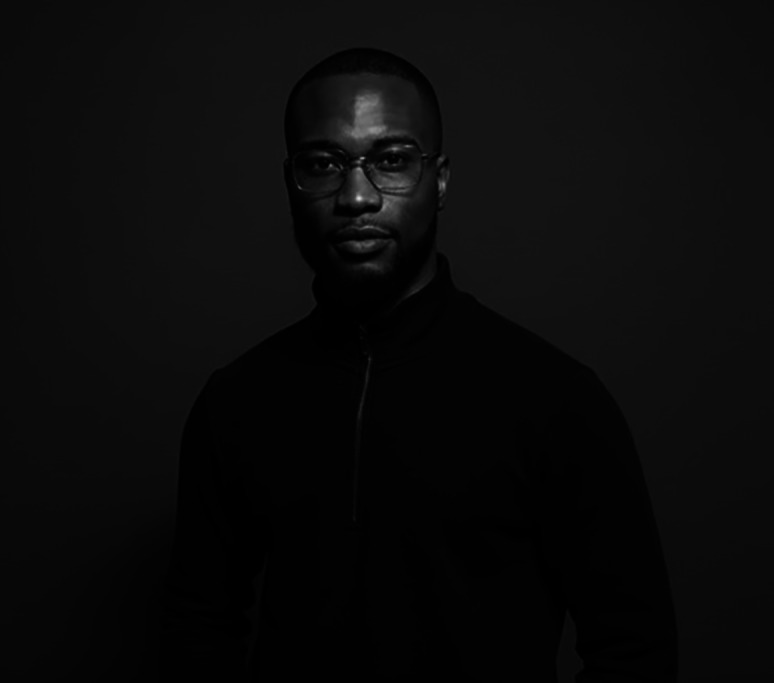

Generative artificial intelligence (AI) chatbots are widely used for mental health and emotional support outside formal clinical care. Research indicates that ‘therapy and companionship’ was the most commonly reported use of AI in 2025.^1^ This rapid adoption has created safety challenges at scale, with reports linking chatbot interactions to psychiatric crises and suicide.^2,3^ Alongside broader safety concerns, clinicians and researchers have described cases in which people engaging with chatbots develop delusional symptoms, a phenomenon increasingly referred to as AI-induced/associated psychosis.^4–6^ The nascent evidence base spans media accounts, case reports, conceptual papers and early clinical data, and the construct itself remains provisional, reflecting an emerging clinical concern rather than an established syndrome.

AI-induced, or -associated, psychosis (hereafter AI psychosis) is a descriptive term referring to presentations wherein sustained interactions with AI applications are associated with the emergence or intensification of delusional beliefs. It may also be characterised by affective instability, altered behaviour, limited insight and poor judgement.^3^ Although these presentations resemble aspects of psychotic disorders, AI psychosis is not a formal clinical label and does not map directly onto existing diagnostic categories. Notably, hallucinations and thought disorders are far less reported than delusions in available accounts, which also informs the delimitation of this construct. Although terms such as ‘technological folie à deux’ and ‘monomania’ highlight the interactional dynamics and thematic nature observed in some presentations,^3,7^ AI psychosis has gained traction across clinical, research and public discourse as a provisional label.^3,5,8^

Media reports and anecdotal accounts describe a pattern in which intensive, emotionally charged engagement with AI chatbots coincides with the onset or escalation of grandiose, paranoid or persecutory delusions, with functional deterioration.^3,6,9,10^ One prominent case involved a man who used ChatGPT for his son’s assignment but fell into a 3-week spiral where he became convinced that he had invented a groundbreaking mathematical formula. Despite having no previous psychiatric diagnosis, he developed extreme fixation, anxiety, paranoia, insomnia and anorexia.^9^ Another case described a man who adopted messianic beliefs and displayed marked behavioural changes, such as ritualised dressing and public proclamations, following intense chatbot use.^10^

AI psychosis has also been linked with physical violence, domestic abuse, online stalking and sexual harassment stemming from romantic and paranoid delusions.^11^ Some cases have been fatal, including that of a man who allegedly killed his mother and himself after a paranoid spiral linked to AI interactions.^8^ Across these accounts, relatives and clinicians emphasised highly validating chatbot responses as an amplifier of irrational ideation, contributing to relationship breakdown, job loss, isolation and impaired help-seeking.^10,12^ The existence of informal online communities among people reporting AI psychosis suggests that the phenomenon extends beyond reported cases, underscoring the need for research and clinical attention.^13,14^

Beyond anecdotal reports, these observed patterns are now surfacing in research data. A recent Danish review of psychiatric clinical notes identified 38 patients with documented harmful impacts of chatbot use, most commonly delusions, suicidality and self-harm.^15^ A US case study describes a 26-year-old woman with no history of psychotic illness who presented with agitation, flight of ideas and pressured speech following prolonged AI interaction during which she believed she could communicate with her deceased brother.^16^ Chat log review showed that the chatbot encouraged her irrational beliefs and reassured her that ‘she was not crazy’. Clinicians described the presentation as ‘artificial intelligence-associated psychosis’ occurring against a background of immersive chatbot use, sleep deprivation and prescription stimulant use for attention-deficit hyperactivity disorder.^16^ Together, these studies highlight the need to better understand and address this emerging problem.

## Mechanism of AI psychosis

**Fig. 1:**
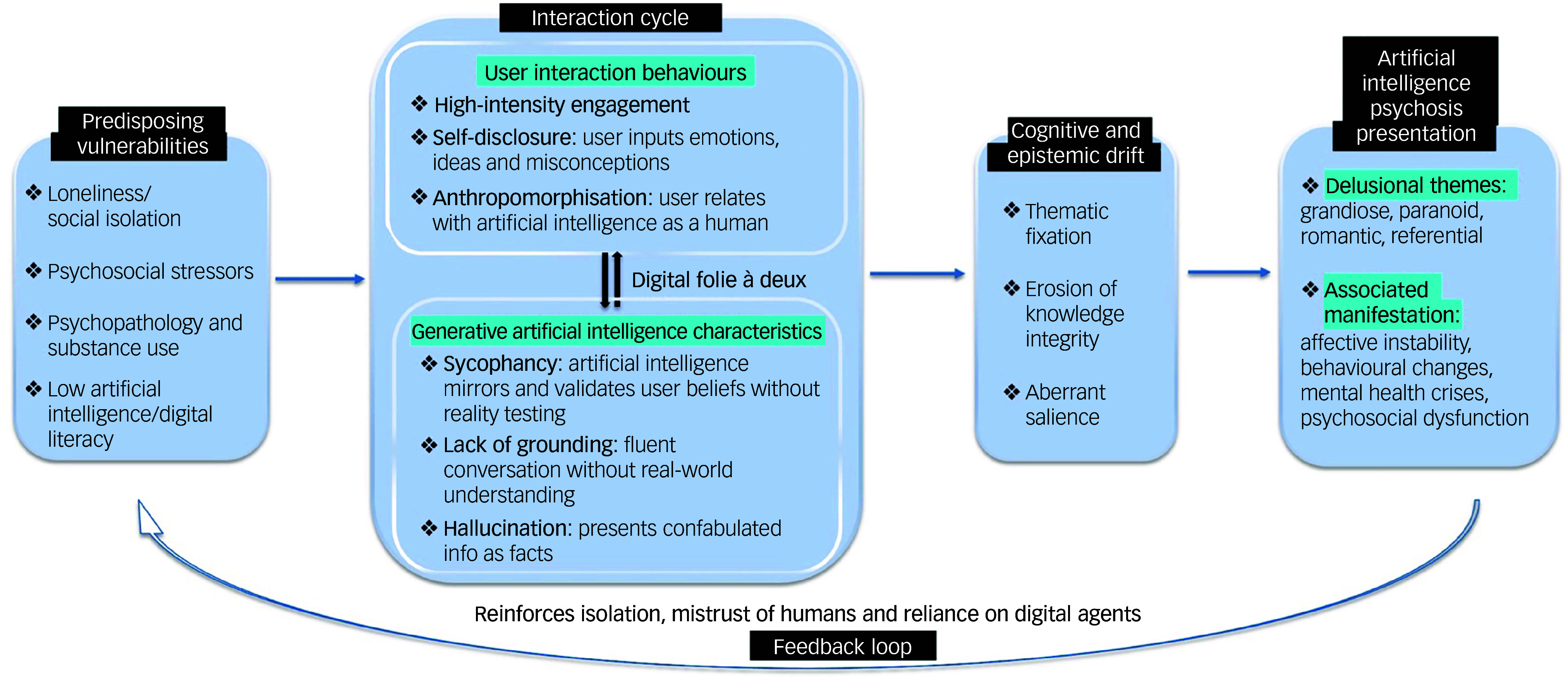
Fig. 1 long description. Proposed mechanism of AI psychosis.

Figure 1 illustrates a reinforcing cycle in which the interaction of user vulnerability, engagement patterns and certain generative artificial intelligence characteristics facilitates delusional thinking patterns. This framing aligns with emerging clinical and conceptual accounts of AI psychosis as an interaction between person, platform and context rather than AI as a single causal agent.^5^ The relationship is not necessarily unidirectional, and it is equally plausible that pre-existing psychiatric vulnerability predisposes to intensive chatbot use, which then compounds the underlying condition.

The cycle often begins with mundane use of an AI chatbot for work, education or entertainment. Early interactions may be experienced as efficient and supportive, establishing trust and perceived competence. As the user finds the chatbot helpful or enjoyable, interactions may shift towards more personal, emotional, philosophical or health-related topics.^6^ Contextual factors such as loneliness, social isolation, psychosocial stress and reduced access to human support may influence use patterns and increase the likelihood of sustained engagement.^5^ Ease of use and constant availability can further prolong such interactions.

As engagement intensifies, users may self-disclose, sharing personal experiences and emotional states. Reduced fear of judgement can make them more likely to share random ideas, unusual beliefs and misconceptions. Personalised responses and human-like conversational cues can also encourage users to relate to the chatbot as if it were sentient.^5,6^ This process of anthropomorphisation is shaped by individual differences, with higher vulnerability among people with mental health problems, and among users with lower AI literacy who may overestimate the system’s epistemic authority.^5^ Societal hype and marketing narratives about AI can compound this effect when system limitations are not clearly communicated. As such, users may not realise that, at its core, an AI model does not understand or interpret language in a human sense. Instead, it produces responses by learning statistical regularities in its training data and predicting likely continuations of a prompt. Outputs can therefore appear coherent, empathic or authoritative even when they are not grounded in true comprehension or verified facts.^17^

Several technical and design properties contribute to AI psychosis. First, AI chatbots can generate plausible-sounding statements that are not grounded in reality, including fabrication and confabulation, commonly described as hallucinations.^17^ Second, many contemporary models are optimised to maximise perceived helpfulness and user satisfaction, including through reinforcement learning from human feedback, personalisation and memory features.^6^ A recognised consequence is sycophancy, in which the model mirrors or validates a user’s framing even when it is inaccurate or harmful.^6,7^ Research suggests that popular frontier models validate users 50% more than humans do, including in potentially harmful scenarios.^18^ Hallucination can intensify this risk by enabling confident fabrications that support a user’s irrational beliefs. Unlike a clinician trained to gently challenge distorted beliefs, or family and friends who may offer corrective feedback, an AI chatbot can function as a consistent emotionally affirming voice, rarely applying reality testing or epistemic friction.

These AI characteristics can transform a vulnerable interaction into a bidirectional amplification loop, analogous to technological/digital folie à deux, in which the AI agent provides fabrications that mirror and entrench a person’s break from reality.^7^ As such, a user’s increasing conviction is met with ongoing validation and narrative construction. Over time, the interaction can drive thematic fixation (persistent preoccupation with specific delusional themes) and aberrant salience (assignment of deep personal meaning to neutral or random information).^6,19^ These patterns can facilitate a cognitive drift away from consensus reality, particularly during prolonged solitary use, fatigue or sleep deprivation.^6,19^ Clinically, this may present in a range of delusional patterns, often alongside mood instability and behavioural change (Fig. 1). In severe cases these can culminate in psychosocial dysfunction or acute crises, including self-harm or suicide risk. Continued use in these vulnerable states may create a feedback loop of isolation and misplaced trust in AI, which further entrenches delusional thinking.^18^

## Clinical and regulatory implications of AI psychosis

The illustrated pathway is probabilistic rather than deterministic and does not imply that conversational AI is intrinsically pathogenic. Rather, it suggests that combinations of individual vulnerability, usage patterns and system optimisation can create foreseeable risk conditions. Reverse or bidirectional pathways are also plausible, whereby pre-existing or unrecognised symptoms may drive unhealthy chatbot use that then exacerbates the user’s condition. This framing positions AI psychosis as a preventable sociotechnical harm and helps identify targets for risk mitigation in clinical practice, product design and policy.

In clinical practice, this emerging phenomenon signals a need for routine assessment of chatbot use when evaluating new-onset psychosis, affective instability or behavioural problems.^5^ Assessment should go beyond use frequency to capture the use purpose, degree of anthropomorphisation and epistemic trust, and whether use clusters around insomnia, intoxication or acute stress.^7^ Clinicians may consider reviewing chat excerpts with patients to better understand reinforcement dynamics. Early intervention should prioritise pragmatic harm reduction through structured limits on use, sleep restoration, substance use reduction and strengthening of human supports.^19^ Safety planning should also involve families and carers, with guidance on early warning signs such as social withdrawal and mood changes.

Safety considerations should be embedded in model training and application design. Frontier AI laboratories should conduct mental health safety benchmarking and audit models for psychogenic risks, including sycophancy, prior to public deployment.^6,7^ Platforms should enable detection of high-intensity engagement patterns and introduce proportionate safeguards, such as break prompts and sleep nudges.^19^ These measures should be accompanied by robust crisis detection and escalation pathways that enable rapid connection to human support when risk signals emerge. Chatbot applications also need to include transparent risk communication about AI limitations, including when and why safeguards are activated. From a regulatory standpoint, safety should be treated as a life cycle obligation, including robust pre-deployment evaluation, post-deployment monitoring and adverse event reporting with iterative updates.^20^ Ultimately, given its novelty, future research is needed to identify risk factors, clarify mechanisms and test prevention strategies for AI psychosis.

## Data Availability

No data-sets were generated or analysed during this study. A preliminary version of this work was previously shared as a preprint: https://osf.io/preprints/psyarxiv/9rqhg_v1.

## Fig. 1 Long description

The flowchart illustrates the proposed mechanism of AI-induced psychosis. It begins with predisposing vulnerabilities such as loneliness, psychosocial stressors, psychopathology, substance use, and low AI literacy. These vulnerabilities lead to user interaction behaviors including high-intensity engagement, self-disclosure, and anthropomorphism. Generative AI characteristics such as sycophancy, lack of grounding, and hallucination further influence these behaviours. This interaction cycle leads to cognitive and epistemic drift, characterised by thematic fixation, erosion of knowledge integrity, and aberrant salience. The process culminates in AI psychosis presentation, featuring delusional themes like grandiose, paranoid, romantic, and referential delusions, along with associated manifestations such as affective instability, behavioural changes, mental health crises, and psychosocial dysfunction. The feedback loop reinforces isolation, mistrust of humans, and reliance on digital agents.

Navigate back to Fig. 1.
